# Perceptions of health professionals in providing care for people with anorexia nervosa and bulimia nervosa: a systematic review and meta-synthesis of qualitative studies

**DOI:** 10.1590/0102-311XEN223122

**Published:** 2023-08-11

**Authors:** Bruna Bortolozzi Maia, Felipe Gonçalves Campelo, Elaine Campos Guijarro Rodrigues, Érika Arantes Oliveira-Cardoso, Manoel Antonio dos Santos

**Affiliations:** 1 Faculdade de Filosofia, Ciências e Letras de Ribeirão Preto, Universidade de São Paulo, Ribeirão Preto, Brasil.

**Keywords:** Mental Health Services, Health Personnel, Feeding and Eating Disorders, Serviços de Saúde Mental, Pessoal de Saúde, Transtornos da Alimentação e da Ingestão de Alimentos, Servicios de Salud Mental, Personal de Salud, Trastornos de Alimentación y de la Ingestión de Alimentos

## Abstract

This study sought to synthesize and reinterpret findings from primary qualitative studies on the experience of health professionals in caring for people with anorexia nervosa and bulimia nervosa. We conducted a systematic review of the literature with the SPIDER search strategy assessing six databases. A meta-synthesis was performed with data from qualitative studies. Two independent reviewers screened and assessed the articles, extracted data from the articles and elaborated thematic synthesis. Nineteen articles met the inclusion/exclusion criteria. The meta-synthesis revealed three descriptive themes: Going outside the comfort zone: hard relational experiences of health professionals in providing care for people with anorexia nervosa and bulimia nervosa; Reflecting on treatment: relevance of discussion, communication, and flexibility in health professionals’ work with anorexia nervosa and bulimia nervosa; and Dealing with ambivalences: experiences of health professionals with family members of people with anorexia nervosa and bulimia nervosa. We elaborated two analytical themes: Making work with eating disorders palatable: malleability necessary for health professionals in bonding with people with anorexia and bulimia nervosa and their families; and Leaving the professional comfort zone: transition from multi to interdisciplinary. Thus, mental health professionals who work with people diagnosed with anorexia and bulimia nervosa cope with hard emotional experiences that makes them feel out of their comfort zone, requiring flexibility to benefit a good therapeutic alliance, but there are still difficulties in promoting interdisciplinarity.

## Resumo

Este estudo buscou sintetizar e reinterpretar os achados de estudos qualitativos primários sobre a experiência de profissionais de saúde no cuidado a pessoas com anorexia e bulimia nervosa. Realizamos uma revisão sistemática da literatura com a estratégia de busca estruturada SPIDER em seis bases de dados. Foi realizada uma metassíntese com dados destes estudos qualitativos. Os artigos foram triados e avaliados por dois revisores independentes que extraíram dados desses artigos e elaboraram uma síntese temática. No total, 19 artigos atenderam nossos critérios de inclusão e exclusão. A metassíntese revelou três temas descritivos: Sair da zona de conforto: profissionais de saúde tiveram vivências relacionais difíceis no cuidado à pessoa com anorexia e bulimia nervosa; Refletir sobre o tratamento: a relevância da discussão, comunicação e flexibilidade no trabalho dos profissionais de saúde; e Lidar com ambivalências: vivências de profissionais de saúde com familiares de pessoas com anorexia e bulimia nervosa. Elaboramos dois temas analíticos: Tornando o trabalho com distúrbios alimentares agradável: maleabilidade necessária para vincular profissionais de saúde à pessoa com anorexia e bulimia nervosa e sua família; e Saindo da zona de conforto profissional: transição do multi para o interdisciplinar. Assim, os profissionais de saúde mental que trabalham com pessoas diagnosticadas com anorexia e bulimia nervosa enfrentam experiências emocionais difíceis que os fazem se sentir fora de sua zona de conforto, exigindo flexibilidade para o benefício de uma boa aliança terapêutica, mas ainda há dificuldades em promover a interdisciplinaridade.

## Resumen

Este estudio buscó sintetizar y reinterpretar los hallazgos de estudios cualitativos primarios sobre la experiencia de profesionales de la salud en el cuidado de personas con anorexia y bulimia nerviosa. Realizamos una revisión sistemática de la literatura con la estrategia de búsqueda estructurada SPIDER en seis bases de datos. Se realizó una metasíntesis con los datos de estos estudios cualitativos. Los artículos fueron seleccionados y evaluados por dos revisores independientes que extrajeron datos de estos artículos y elaboraron una síntesis temática. En total, 19 artículos cumplieron con nuestros criterios de inclusión y exclusión. La metasíntesis reveló tres temas descriptivos: Salir de la zona de confort: los profesionales de la salud tuvieron vivencias relacionales difíciles en el cuidado a la persona con anorexia y bulimia nerviosa; Reflexionar sobre el tratamiento: la relevancia de la discusión, la comunicación y la flexibilidad en el trabajo de los profesionales de la salud; y Lidiar con las ambivalencias: experiencias de los profesionales de la salud con familiares de personas con anorexia y bulimia nerviosa. Elaboramos dos temas analíticos: Hacer agradable el trabajo con los trastornos alimentarios: maleabilidad necesaria para vincular a los profesionales de la salud con la persona con anorexia y bulimia nerviosa y su familia; y Salir de la zona de confort profesional: transición de lo múltiple a lo interdisciplinario. Por lo tanto, los profesionales de la salud mental que trabajan con personas diagnosticadas con anorexia y bulimia nerviosa enfrentan experiencias emocionales difíciles que los hacen sentir fuera de su zona de confort, lo que requiere flexibilidad en beneficio de una buena alianza terapéutica, pero aún existen dificultades para promover la interdisciplinariedad.

## Introduction

This systematic review and metasynthesis sought to synthesize and reinterpret results of primary qualitative studies about the experience of caring for people with eating disorders from the perspective of health professionals. People diagnosed with eating disorders present significant alterations in their eating behavior pattern ^1^ ^,^ ^2^ ^,^ ^3^. Two subtypes stand out due to great social visibility: anorexia nervosa and bulimia nervosa. The main characteristics described in both cases are significant changes in eating behavior in order to avoid weight gain, accompanied by intense distortion of body image ^4^.

Due to the severity and persistence of symptoms, the specialized literature recommends that people with eating disorders should receive intense and continuous treatment conducted by professionals such as doctors, psychologists, nutritional, psychiatrists, among other health professionals. Thus, it is recommended that such therapeutics should be carried out by a multidisciplinary team with interdisciplinary practices ^5^ ^,^ ^6^. In addition to dictating a team composition with different specializations, an interdisciplinary approach is one in which different knowledge areas can establish exchanges and dialogues, implementing a specific therapeutic plan for each patient ^7^. Furthermore, care for people with eating disorders should be conducted with family members ^8^, and coordinated with other health services (general, social, or specialized services), promoting comprehensive care for the population ^9^, along with an attitude that considers the social context in health practices ^10^.

Despite extensive research in the area, high rates of non-compliance and difficulties in adhering to specialized treatment still prevail ^11^ ^,^ ^12^ ^,^ ^13^ ^,^ ^14^. The tenacity with which people with anorexia and bulimia nervosa resist therapeutic strategies may be related to the failure to recognize eating disorder as a problem, since restrictive eating patterns can be experienced as a lifestyle. Studies also indicate that these people may have additional difficulties in forming and maintaining emotional relationships with their family members, friends, as well as with healthcare professionals themselves ^7^ ^,^ ^15^ ^,^ ^16^.

Research shows that therapeutic alliance is one of the most important variables related to treatment outcome, especially in anorexia nervosa cases ^16^ ^,^ ^17^. Despite recognizing the importance of therapeutic alliance for treatment, health professionals face difficulties in forming this relationship, which often makes them feel frustrated, hopeless, and incompetent in facing the persistence of symptoms ^18^ ^,^ ^19^.

Working with anorexia nervosa/bulimia nervosa people can aggravate stressful situations, which make health professionals a vulnerable population regarding mental health ^20^. This is because doctors, nurses, and other health workers are subjected to situations of exhaustion and intense work for a long period of time, extreme situations linked to death and finitude, among other factors that can increase the risk of mental illness ^20^ ^,^ ^21^. Professionals who work with eating disorders are subjected to several challenging moments in their work, which can even compromise their emotional availability in caring for patients ^12^ ^,^ ^22^, which is essential when caring for people with these diagnoses ^16^ ^,^ ^17^.

Therefore, knowing that the scientific evidence is about how health professionals perceive and experience care for people with eating disorders can provide important clues for understanding the potentials and difficulties encountered when establishing a therapeutic bond. This knowledge can provide subsidies to refine treatment planning, as well as strengthen the mental health and well-being of professionals. Metasyntheses have already been published about the experience of patients in treatment ^23^ ^,^ ^24^, as well as family members who accompany them in health services ^25^ ^,^ ^26^ ^,^ ^27^. However, a consistent review of qualitative studies regarding the experience of health professionals in the care provided to people with anorexia nervosa/bulimia nervosa has not yet been conducted. We expect that systematically gathering qualitative evidence from selected studies following a rigorous methodological process can promote a new conceptual understanding of the summarized results, transcending previous results and allowing us to formulate new understandings ^28^.

In view of the above, this study aims to synthesize and reinterpret the results from primary qualitative studies about the experience of caring for people with anorexia nervosa/bulimia nervosa from the perspective of health professionals.

## Method

### Design

The systematic research trajectory followed 10 steps: (1) Elaboration of the research question guided by the SPIDER strategy; (2) Definition of selection and exclusion criteria, and choice of appropriate databases for the research area; (3) Elaboration of the search strategy based on specific descriptors for each database; (4) Searching the databases, with validation by another researcher who independently evaluated the information; (5) Screening and selection from titles and abstracts, also comparing the results with the second independent reviewer and using the Rayyan tool (https://www.rayyan.ai/); (6) Calculation of the kappa index of inter-rater agreement; (7) Reading the selected articles in full and final selection of the analysis corpus; (8) Qualitative analysis of the methodological procedures of the studies studied based on the Critical Appraisal Skills Program (CASP) ^29^; (9) Coding the results of selected articles using the QDAMiner 9.0 Lite program (https://provalisresearch.com/); (10) Description and analysis of the material.

This study was registered on the PROSPERO platform ^30^ (under the protocol CRD42022311740). The Enhancing Transparency in Reporting the Synthesis of Qualitative Research (ENTREQ) guide was used to report the essential elements that must compose a qualitative evidence synthesis ^31^.

### Research question, eligibility criteria, and research strategy

The SPIDER strategy ([S] sample; [PI] phenomenon of interest; [D] study design; [E] evaluation; [R] research type) was chosen because it is considered an adequate tool for reviewing studies with qualitative methods, providing greater rigor to the research ^32^. Thus, the following research guiding question was elaborated based on the SPIDER strategy: What is the qualitative evidence available in the literature about the experience of health professionals in the care of people with eating disorders?

Eligibility criteria were defined as follows: Inclusion criteria: (a) primary qualitative studies; (b) studies consistent with the research question developed with the SPIDER strategy; (c) articles that include the perception of health professionals, trainees, or workers in the Results section as a category or subcategory. Exclusion criteria: (a) qualitative, mixed, secondary, literature review, or theoretical-reflective studies; (b) gray literature, such as theses, dissertations, monographs, books, or chapters; (c) letter to the editor, editorial, commentary, opinion articles, and abstracts; (d) studies that do not include health professionals (only patients and/or their families); (e) studies published in languages ​​other than Portuguese, English, Spanish, or French; (f) studies regarding eating disorders that do not meet the diagnostic criteria for anorexia or bulimia.

After defining the databases regarding their relevance to the ​​knowledge area and seeking to encompass the national and international research scenario, the search terms were selected, which meant choosing the appropriate descriptors for each database. The SPIDER search strategy was defined by combining the descriptors of each acronym, suitable for each database. No date restriction was applied. The search used the Boolean operators OR between the descriptors of the same acronym and AND between each one of them, as follows: (S1 OR S2 OR Sn...) AND (Pi1 OR Pi2 OR Pin...) AND (D1 OR D2 OR Dn...) AND (E1 OR E2 OR En...) AND (R1 OR R2 OR Rn...). The “advanced search” tool was used in the databases. A track of descriptors assembled from DeCS/MeSH is shown in Supplementary Material 1 (https://cadernos.ensp.fiocruz.br/static//arquivo/suppl-1-e00223122_2723.pdf).

### Study search and selection

The study based on the described strategy was conducted by two reviewers independently in February 2022 in six databases: LILACS, PsycINFO, PubMed, CINAHL, Scopus, and Web of Science. A total of 3,247 articles were identified in this first stage. From this first sieve, the selection of articles was refined with the support of the Rayyan software for systematic reviews ^33^. The use of a software makes the study selection process more transparent and reliable ^34^. Duplicate articles were excluded (n = 55). In the next phase, the same independent reviewers applied the eligibility criteria described above. The two reviewers blindly selected the articles they considered to be included or excluded. After this step, removing the blinding of the Rayyan tool, it was possible to see the concordances and disagreements between the reviewers.

**Figure 1:**
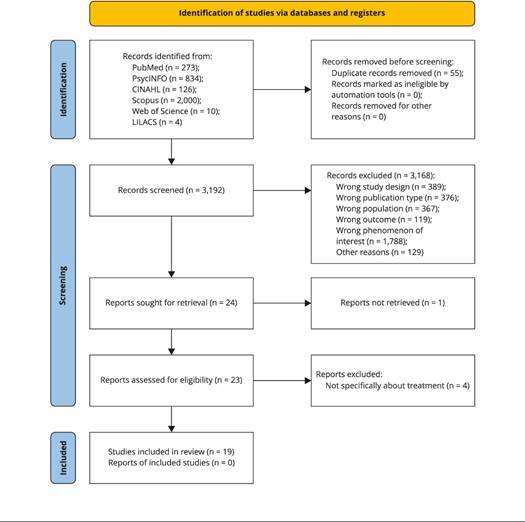
PRISMA (Preferred Reporting Items for Systematic Reviews and Meta-Analyses) diagram of study selection process.

The kappa index calculation was performed to ensure a sufficiently good agreement rate between reviewers ^35^. We obtained a value of 0.825 herein, demonstrating excellent agreement between the lists produced by the evaluators. As a result of this process, 23 articles met the eligibility criteria. Figure 1 shows the number of materials excluded according to each criterion. Thus, we proceeded to the next step: full reading of the recovered materials. The two reviewers read the remaining articles in full, except for one which was only published in full in Japanese. After the full reading, the two reviewers discussed again the eligibility of each article. From the agreement of the reviewers, four articles were also excluded at this stage. The references that were excluded after their full text was reviewed and the reason for exclusion is shown in Supplementary Material 2 (https://cadernos.ensp.fiocruz.br/static//arquivo/suppl-2-e00223122_9940.pdf). Therefore, the final corpus of this metasynthesis consisted of 19 articles.

The selection and exclusion process of articles is described in the flowchart (Figure 1) developed from the PRISMA strategy (Preferred Reporting Items for Systematic Reviews and Meta-Analyses) ^36^.

The data from the 19 studies were described based on extracting the following variables: first author, year of publication, country where the study was conducted, the study objective, design, data collection and analysis forms, as well as the number and characteristics of participants (Box 1).

**Box 1:** Features of the included studies (n = 19).

STUDY (YEAR/COUNTRY)	AIM	METHODS	n	PROFESSIONAL CHARACTERIZATION
Webb et al. ^38^ (2022/England)	To explore clinicians’ perspectives and experience of supporting adults with severe anorexia nervosa in intensive treatment settings and the opportunities and challenges associated with these	Semi-structured interviews; Thematic content analysis	21	6 consultant psychiatrist, 3 occupational therapist, 1 clinical service manager, 2 nurse specialist, 2 nurse therapist, 1 dietician, 2 counselling psychologist, 1 mental health nurse, 2 assistant psychologist, 1 day unit manager Years of experience: 1 to 10+
Kanakam ^39^ (2021/England)	To explore therapists’ perspectives on how ethnic minority females diagnosed with eating disorders access specialist eating disorder services and what are the therapists’ experiences of working with ethnic minority females diagnosed with eating disorders in these services	Semi-structured interviews; Thematic analysis and critical realist epistemology	11	10 clinical/counselling psychologists; 1 family therapist Years of experience: 2.5 to 16
McDonald et al. ^40^ (2021/England)	To examine the face validity of Khalsa et al. recovery criteria with service users and eating disorder therapists	Semi-structured interviews; Thematic content analysis	8	NHS adult eating disorder service and one charitable adult eating disorder service Years of experience: not specified
Webb & Schmidt ^41^ (2021/England)	To explore the clinicians’ perspective of the barriers and facilitators to supporting students with eating disorders transitioning to university	Semi-structured interviews; Thematic content analysis	12	6 clinical psychologist, 1 clinical service manager, 1 counselling psychologist, 1 mental health nurse, 1 occupational therapist, 1 consultant psychiatrist, 1 clinical nurse specialist 90.8% female Years of experience: not specified
Wu & Chen ^42^ (2021/Taiwan)	To explore nurses’ perceptions on and experiences in conflict situations in caring for adolescents with anorexia nervosa	Semi-structured interviews; Thematic content analysis	10	Nurses form a general pediatric ward 100% female Years of experience: 2 to 14
Farrington et al. ^43^ (2020/Ireland)	To explore mental health nursing students’ experiences of working with adolescents who are receiving inpatient treatment for an eating disorder	In-deep interviews; Schematic content analysis	4	Final-year student mental health nurses 100% remale Years of experience: < 1 to 1
Davidson et al. ^44^ (2019/Australia)	To explore the considerations that influence the medical decisions of physicians when treating patients with eating disorders in the acute setting	Semi-structured interviews; Qualitative content analysis	10	3 consultants doctors, 2 registrars, 1 resident, 4 interns Years of experience: < 1 to 16
Dimitropoulos et al. ^45^ (2019/Canada)	To identify how FBT practitioners applied FBT for atypical anorexia nervosa for adolescents in their clinical practice, and if there were any implementation challenges and adaptations to the model for this population	Semi-structured interviews; Qualitative content analysis	23	FBT practitioners in public and private practice Years of experience: 1 to 16
Holmes ^46^ (2018/England)	To explore health professional views on the role of sociocultural perspectives in eating disorder treatment, with a particular focus on cultural constructions of femininity	Semi-structured interviews; Thematic discourse analysis	12	3 therapists and counsellors, 2 psychologists, 1 psychiatrist, 1 nurse, 1 occupational therapist, 4 supportive health professionals 90.8% female Years of experience: not specified
Kinnaird et al. ^47^ (2018/England)	To explore clinicians’ views on whether men have gender-specific treatment needs, and how far these needs require treatment adaptations	In-deep interviews; Framework analysis	10	Clinicians currently working within the outpatient and day-care teams treating adults with eating disorders 100% female Years of experience: 3 (minimum)
Watt & Dickens ^48^ (2018/Australia)	To explore mental health clinicians perspectives on community mealtime management with children and adolescents diagnosed with an eating disorder	Interviews with open-ended questions; Thematic content analysis	6	1 consultant psychiatrist, 1 clinical nurse specialist, 1 nurse therapist, 2 staff nurses, 1 support worker Years of experience: 2 to 15
Wehrens & Walters ^49^ (2018/The Netherlands)	To investigate the experiences of patients and professionals about the ability of health-care professionals to understand the lived experiences of their patients	Imitation game; Focus groups; Thematic content analysis	6	Therapists from a center with outpatient treatment for eating disorders 90% female Years of experience: 12 to 25
Harken et al. ^50^ (2017/United States)	To describe the perceptions of inpatient pediatric hospitalist physicians, registered nurses, and care assistants at a tertiary pediatric hospital regarding caring for children with eating disorders who are hospitalized for medical stabilization	Semi-structured interviews; Thematic content analysis	20	Pediatric hospitalist physicians, registered nurses, and care assistants 85% female Years of experience: < 1 to 7+
Kinnaird et al. ^51^ (2017/England)	To explore the experiences of clinicians working with comorbid anorexia nervosa and autism spectrum disorder	Semi-structured interviews; Qualitative content analysis	9	Nurse therapists, cognitive behavioral therapists, a cognitive analytical therapist, a psychotherapist, a dietician, an occupational therapist 100% female Years of experience: 3 (minimum)
Cruzat et al. ^52^ (2013/Spain)	To describe the aspects identified as facilitators in order to achieve a positive therapeutic alliance from the perspective of anorexic patients and their positive therapeutic alliance from the perspective of anorexic patients and their therapists	Relational-descriptive study; In-deep interview; Grounded theory	2	Therapists of two patients diagnosed with anorexia nervosa Years of experience: not specified
Dimitropoulos et al. ^53^ (2013/Canada)	To identify how FBT practitioners applied FBT for atypical anorexia nervosa for adolescents in their clinical practice, and if there were any implementation challenges and adaptations to the model for this population	Fundamental qualitative description; Semi-structured interview; Thematic content analysis	23	Practitioners of family-based treatment with adolescents with atypical anorexia nervosa Years of experience: 3 to 19
Hunt & Churchill ^54^ (2013/England)	To identify general practitioners’ understandings and experiences of diagnosing and managing patients with anorexia in primary care	Case-based focus groups; Linguistic and discourse analysis	12	General practitioners 50% female Years of experience: mean of 12.8
Dimitropoulos et al. ^55^ (2012/Canada)	To conduct qualitative research on the perspectives of service providers regarding the transition process from pediatric to adult specialized eating disorder tertiary care programs	Focus groups; Qualitative interviews; Grounded theory	18	Occupational therapists, social workers, pediatricians specializing in adolescent medicine, dieticians, nurses, social workers, psychiatrist, transition worker, front-line nurses Years of experience: 2 to 28
Reid et al. ^56^ (2010/Scotland)	To examine healthcare professional’s perspectives of eating disorder’s patients and services	Semi-structured interviews; Thematic content analysis	18	Psychiatrist, psychologist occupational therapist, general practitioner, dietician, dietician manager, endocrinologist Years of experience: not specified

FBT: family-based treatment; NHS: National Health System (United Kingdom).

Source: prepared by the authors.

### Methodological quality of the articles

The *CASP Qualitative Checklist* was used to qualitatively evaluate the methodological properties of the studies. It is a checklist which considers the description and relevance of the objectives, adequacy of the methodological choice and study design, the description of the data collection and analysis strategy, if the ethical procedures were well described and if the data were presented in a sufficiently satisfactory manner ^29^. The analysis of these aspects was performed by two independent reviewers. The two lists were subsequently compared, and disagreements could be discussed until consensus was established. As described in Box 2, all studies presented their objectives clearly, and these proved to be appropriate for qualitative studies. Despite this, most of the retrieved and analyzed articles did not explicitly discuss the researchers’ implication and relationship with the participants.

**Box 2:** Quality appraisal of included studies according to the Critical Appraisal Skills Program (CASP).

STUDY (YEAR)	1	2	3	4	5	6	7	8	9	10
Webb et al. ^38^ (2022/England)	Yes	Yes	Yes	Yes	Yes	Can’t tell	Yes	Yes	Yes	Yes
Kanakam ^39^ (2021/England)	Yes	Yes	Yes	Yes	Yes	Yes	Yes	Yes	Yes	Yes
McDonald et al. ^40^ (2021/England)	Yes	Yes	Yes	Yes	Yes	Yes	Yes	Yes	Yes	Yes
Webb & Schmidt ^41^ (2021/England)	Yes	Yes	Yes	Yes	Yes	Yes	Yes	Yes	Yes	Yes
Wu & Chen ^42^ (2021/Taiwan)	Yes	Yes	Yes	Yes	Yes	Can’t tell	Yes	Yes	Yes	Yes
Farrington et al. ^43^ (2020/Ireland)	Yes	Yes	Yes	Yes	Yes	Can’t tell	Yes	Yes	Yes	Yes
Davidson et al. ^44^ (2019/Australia)	Yes	Yes	Yes	Yes	Yes	Can’t tell	Yes	Yes	Yes	Yes
Dimitropoulos et al. ^45^ (2019/Canada)	Yes	Yes	Yes	Yes	Yes	Can’t tell	Yes	Yes	Yes	Yes
Holmes ^46^ (2018/England)	Yes	Yes	Yes	Yes	Yes	Can’t tell	Yes	Yes	Yes	Yes
Kinnaird et al. ^47^ (2018/England)	Yes	Yes	Yes	Yes	Yes	Yes	Yes	Yes	Yes	Yes
Watt & Dickens ^48^ (2018/Australia)	Yes	Yes	Yes	Yes	Yes	Can’t tell	Yes	Yes	Yes	Yes
Wehrens & Walters ^49^ (2018/The Netherlands)	Yes	Yes	Yes	Can’t tell	Yes	Can’t tell	Yes	Yes	Yes	Yes
Harken et al. ^50^ (2017/United States)	Yes	Yes	Yes	Yes	Yes	Can’t tell	Yes	Yes	Yes	Yes
Kinnaird et al. ^51^ (2017/England)	Yes	Yes	Yes	Yes	Yes	Can’t tell	Yes	Yes	Yes	Yes
Cruzat et al. ^52^ (2013/Spain)	Yes	Yes	Yes	Can’t tell	Yes	Can’t tell	Can’t tell	Yes	Yes	Yes
Dimitropoulos et al. ^53^ (2013/Canada)	Yes	Yes	Yes	Yes	Yes	Can’t tell	Yes	Yes	Yes	Yes
Hunt & Churchill ^54^ (2013/England)	Yes	Yes	Yes	Yes	Yes	Can’t tell	Yes	Yes	Yes	Yes
Dimitropoulos et al. ^55^ (2012/Canada)	Yes	Yes	Yes	Yes	Yes	Can’t tell	Yes	Yes	Yes	Yes
Reid et al. ^56^ (2010/Scotland)	Yes	Yes	Yes	Yes	Yes	Yes	Yes	Yes	Yes	Yes

Source: prepared by the authors.

Note (questions): 1 - Was there a clear statement of the aims of the research?; 2 - Is a qualitative methodology appropriate?; 3 - Was the research design appropriate to address the aims of the research?; 4 - Was the recruitment strategy appropriate to the aims of the research?; 5 - Were the data collected in a way that addressed the research issue?; 6 - Has the relationship between researcher and participants been adequately considered?; 7 - Have ethical issues been taken into consideration?; 8 - Was the data analysis sufficiently rigorous?; 9 - Is there a clear statement of findings?; 10 - How valuable is the research?

### Data analysis

Thematic analysis was performed according to the following methodology ^37^: (1) Reading the selected studies in full using the QDAMiner Lite software to perform the coding line by line; (2) Grouping the codes into similar themes, called descriptive themes, which describes the results of the analyzed primary studies; (3) Development of the analytical theme: at this stage, new interpretations and constructs are elaborated from the original data. The analytical topic is discussed and validated with the help of the research group, to which the researchers are linked.

## Results

The 19 articles analyzed ^38^ ^,^ ^39^ ^,^ ^40^ ^,^ ^41^ ^,^ ^42^ ^,^ ^43^ ^,^ ^44^ ^,^ ^45^ ^,^ ^46^ ^,^ ^47^ ^,^ ^48^ ^,^ ^49^ ^,^ ^50^ ^,^ ^51^ ^,^ ^52^ ^,^ ^53^ ^,^ ^54^ ^,^ ^55^ ^,^ ^56^ were published between 2022 and 2010, showing that the phenomenon studied is relatively new in the specialized literature. More than 60% (n = 12) ^38^ ^,^ ^39^ ^,^ ^40^ ^,^ ^41^ ^,^ ^42^ ^,^ ^43^ ^,^ ^44^ ^,^ ^45^ ^,^ ^46^ ^,^ ^47^ ^,^ ^48^ ^,^ ^49^ of them were published in the last five years. Most studies were carried out in England (n = 8) ^38^ ^,^ ^39^ ^,^ ^40^ ^,^ ^41^ ^,^ ^46^ ^,^ ^47^ ^,^ ^51^ ^,^ ^54^. Three are from Canada ^45^ ^,^ ^53^ ^,^ ^55^ and two from Australia ^44^ ^,^ ^48^. The others were conducted in Ireland ^43^, Taiwan ^42^, The Netherlands ^49^, United States ^50^, Spain ^52^, and Scotland ^56^. Therefore, the corpus of this study does not include Latin American articles, despite the inclusion of a database from this region (LILACS).

Regarding data collection, we observed that most studies were conducted through individual interviews characterized by in-depth, semi-structured, and open questions (n = 17) ^38^ ^,^ ^39^ ^,^ ^40^ ^,^ ^41^ ^,^ ^42^ ^,^ ^43^ ^,^ ^44^ ^,^ ^45^ ^,^ ^46^ ^,^ ^47^ ^,^ ^48^ ^,^ ^50^ ^,^ ^51^ ^,^ ^52^ ^,^ ^53^ ^,^ ^56^. Only three studies used focus groups ^49^ ^,^ ^54^ ^,^ ^55^. In examining the methodological strategies used for data analysis, most studies used thematic content analysis (n = 10) ^38^ ^,^ ^39^ ^,^ ^40^ ^,^ ^41^ ^,^ ^42^ ^,^ ^48^ ^,^ ^49^ ^,^ ^50^ ^,^ ^53^ ^,^ ^56^ and its variants, such as schematic content analysis (n = 1) ^43^ and qualitative content analysis (n = 3) ^44^ ^,^ ^45^ ^,^ ^51^. Moreover, discourse analysis (n = 2) ^46^ ^,^ ^54^, framework analysis (n = 1) ^47^, and grounded theory (n = 2) ^52^ ^,^ ^55^ were also used.

A total of 235 participants composed the total sample of this metasynthesis, including the 19 studies. The samples included several specializations in the health area, such as: clinical service managers, psychiatrists, consultant doctors, general practitioners, endocrinologists, occupational therapists, nurses, dieticians, counseling psychologists, clinical psychologists, social workers, and family therapists. These participants had anywhere from 28 years of experience to almost none (residents and undergraduate, intern, and final-year nursing school students and residents).

The thematic synthesis process revealed 42 codes referring to the results of the 19 studies. These were synthesized and described in three descriptive themes: (1) Going outside the comfort zone: hard relational experiences of health professionals in providing care for people with anorexia nervosa/bulimia nervosa; (2) Reflecting on treatment: relevance of discussion, communication and flexibility in health professionals’ work with anorexia nervosa/bulimia nervosa; (3) Dealing with ambivalences: experiences of health professionals with family members of people with anorexia nervosa/bulimia nervosa in the therapeutic context. From this, two analytical themes were developed: (1) Making work with eating disorders palatable: malleability necessary for health professionals in bonding with people with anorexia nervosa/bulimia nervosa and their families; (2) Leaving the professional comfort zone: transition from multi to interdisciplinary (Box 3).

**Box 3:** Codes and themes generated by the process of thematic synthesis.

INITIAL CODES (LINE-BY-LINE CODING)	DESCRIPTIVE THEMES	ANALYTICAL THEMES
Anxiety/Fear	Going outside the comfort zone: hard relational experiences of health professionals in providing care for people with anorexia nervosa/bulimia nervosa	ANALYTICAL THEME 1: Making work with eating disorders palatable: malleability necessary for health professionals in bonding with people with anorexia nervosa/bulimia nervosa and their families ANALYTICAL THEME 2: Leaving the professional comfort zone: transition from multi to interdisciplinary
Frustration		
Doubt		
Difficulty		
Lack of knowledge		
Hope		
Empathy		
Valuing the promotion of bond		
Difficulty in therapeutic alliance		
Difficulty in communication		
Difficulty in expressing feelings		
Denial of the disease		
Non-engagement in treatment		
Negative feelings from patients		
Relapse in treatment		
Complexity of the disease		
Tube feeding		
Intensive care		
Increase in cases		
Physical health		
Mental health		
Loneliness		
Social aspects		
Medical support		
Flexibility/Individuality		
Responsibility and commitment		
Multidisciplinary team - importance	Reflecting on treatment: relevance of discussion, communication, and flexibility in health professionals’ work with anorexia nervosa/bulimia nervosa	
Impasses in teamwork		
Communication between different services		
Lack of time		
Lack of resources		
Planning/Protocol		
Knowledge/Experience		
Therapeutic groups		
Support from other people diagnosed		
Develop skills		
No separation between body/mind		
Weight gain		
Feeding		
Social support	Dealing with ambivalences: experiences of health professionals with family members of people with anorexia nervosa/bulimia nervosa in the therapeutic context	
Importance of family members		
Difficulties of family members		

Source: prepared by the authors.

### Theme 1: Going outside the comfort zone: hard relational experiences of health professionals in providing care for people with anorexia nervosa/bulimia nervosa

This theme highlights how the therapeutic relationship between professionals and patients is experienced, as well as the emotional experiences of the professionals. Despite being valued, the therapeutic alliance proves to be difficult for the participants of the retrieved studies. These obstacles are related to the complex and multifactorial characteristics of eating disorders ^38^ ^,^ ^40^ ^,^ ^42^ ^,^ ^43^ ^,^ ^44^ ^,^ ^45^ ^,^ ^46^ ^,^ ^47^ ^,^ ^48^ ^,^ ^50^ ^,^ ^54^ ^,^ ^55^ ^,^ ^56^, the non-recognition of eating disorder as a disease ^39^ ^,^ ^41^ ^,^ ^42^ ^,^ ^43^ ^,^ ^44^ ^,^ ^45^ ^,^ ^47^ ^,^ ^54^ ^,^ ^55^, and the difficulty of communicating with patients ^42^ ^,^ ^43^ ^,^ ^44^ ^,^ ^47^ ^,^ ^49^ ^,^ ^50^ ^,^ ^51^ ^,^ ^54^. From the perspectives of the people studied, this scenario causes feelings such as: doubt, frustration, incompetence, anxiety, and fear ^38^ ^,^ ^39^ ^,^ ^41^ ^,^ ^42^ ^,^ ^43^ ^,^ ^44^ ^,^ ^48^ ^,^ ^49^ ^,^ ^50^ ^,^ ^54^ ^,^ ^55^. The professionals reinforced the importance of engaging the person with anorexia nervosa/bulimia nervosa in the treatment. When there is a good link between professional and patient, the studies highlight the professional’s openness to feelings of empathy, improving the quality of care offered ^38^ ^,^ ^40^ ^,^ ^43^ ^,^ ^50^ ^,^ ^52^ ^,^ ^56^.

Despite the difficulties in establishing a therapeutic alliance, these represents an important aspect for professionals in caring for people with eating disorder. The retrieved studies highlight ^38^ ^,^ ^39^ ^,^ ^43^ ^,^ ^49^ ^,^ ^50^ ^,^ ^51^ ^,^ ^52^ ^,^ ^53^ ^,^ ^56^ the relationship between health workers and patients during the course of treatment in the sense of promoting a welcoming posture: “*a relational space that allows for the development of a positive bond*” ^52^ (p. 178), causing the professional to care for the development of a “*warm* [therapeutic] *relationship*” ^38^ (p. 5).

However, many emphasize that there are several obstacles to forming and maintaining a good interpersonal relationship with patients ^38^ ^,^ ^39^ ^,^ ^42^ ^,^ ^43^ ^,^ ^50^ ^,^ ^51^ ^,^ ^55^ ^,^ ^56^. Some professionals argue that this difficulty in forming and maintaining a good therapeutic relationship is related to the characteristics of anorexia nervosa/bulimia nervosa, which make contact and communication with these patients be considered “difficult” ^42^ ^,^ ^43^ ^,^ ^46^ ^,^ ^48^ ^,^ ^50^ ^,^ ^51^ ^,^ ^52^ ^,^ ^53^ ^,^ ^54^, and “*outside their comfort zone*” ^44^ (p. 3).

The impasses in establishing a therapeutic relationship with the person with anorexia nervosa/bulimia nervosa in some studies are linked to the need to convince them to do something that goes against their own will, as is evident in the following report: “*you’re making these kids do something that absolutely horrifies them. I saw this as very... very difficult and upsetting*” ^43^ (p. 685). This idea is also related to the patients’ lack of motivation with the treatment ^54^ ^,^ ^55^ ^,^ ^56^, making it “*difficult to persuade patients to accept specialist care*” ^54^ (p. 463).

Thus, from the perspective of the professionals participating in the recovered studies, the attitude of patients in the services which makes the therapeutic relationship most difficult is the denial of eating disorder as a disease ^39^ ^,^ ^41^ ^,^ ^42^ ^,^ ^43^ ^,^ ^44^ ^,^ ^45^ ^,^ ^47^ ^,^ ^54^ ^,^ ^55^, as in the following report of a professional: “*It can be challenging with the patients who don’t have the insight into their illness, because they don’t necessarily want your help*” ^44^ (p. 6). As a result, patients are described by participants in several studies ^38^ ^,^ ^39^ ^,^ ^42^ ^,^ ^43^ ^,^ ^44^ ^,^ ^45^ ^,^ ^48^ ^,^ ^53^ ^,^ ^54^ ^,^ ^55^ as non-collaborative and manipulative. A practitioner summarizes the content of contact with patients as follows: “*I found it hard...*” ^46^ (p. 556).

The selected studies also suggest that the difficulty in communicating with people with eating disorders may be a factor that hinders establishing a therapeutic alliance. Participants state that there is little emotional openness, distrust, and little ability to express feelings ^42^ ^,^ ^43^ ^,^ ^44^ ^,^ ^47^ ^,^ ^49^ ^,^ ^50^ ^,^ ^51^ ^,^ ^54^. The narrative of nurses participating in one of the studies highlights that not establishing eye contact is a non-verbal element that underlines the emotional closure of patients: “*She speaks coldly and doesn’t even look at me*” ^42^ (p. 1390). Studies suggest that there is a difficulty in establishing effective communication, which raises doubts in participating professionals about the best way to talk to patients ^43^ ^,^ ^46^ ^,^ ^51^. A professional expressly questioned herself: “*God, what if I say the wrong thing to the service user?*” ^43^ (p. 684). In view of this, survey participants reported feeling nervous: “*I used to be quite nervous about saying anything*” ^39^ (p. 426).

Others also reported that professionals have doubts when making decisions about treatment ^38^ ^,^ ^39^ ^,^ ^40^ ^,^ ^41^ ^,^ ^42^ ^,^ ^43^ ^,^ ^44^ ^,^ ^46^ ^,^ ^47^ ^,^ ^48^ ^,^ ^54^, generating several insecurities regarding their own abilities, as in the following reports: “*Am I doing the right thing?*” ^48^ (p. 34). In other investigations ^41^ ^,^ ^44^ ^,^ ^45^ ^,^ ^46^ ^,^ ^50^ ^,^ ^56^ professionals felt they did not have enough knowledge to work with eating disorders, they needed to read more studies or receive specialized training in the area.

In addition, some of the retrieved studies revealed that professionals felt worried and anxious at times of relapse and worsening of the patient’s condition ^38^ ^,^ ^39^ ^,^ ^40^ ^,^ ^41^ ^,^ ^44^ ^,^ ^47^ ^,^ ^48^ ^,^ ^50^ ^,^ ^54^. One professional ponders: “*but if the person is far away and then, suddenly, they deteriorate severely, then it would be very difficult*” ^41^ (p. 448). Although concerned about the worsening of the clinical picture, a patient’s improvement also always seems very fragile, being anxiogenic for health professionals: “*It could just take the littlest thing for you to say to completely regress in their recovery*” ^43^ (p. 685), bringing anguish to professionals: “*There’s and anxiety there about holding that risky responsibility*” ^38^ (p. 6).

Another prevalent feeling in these moments concerns frustration ^38^ ^,^ ^39^ ^,^ ^42^ ^,^ ^44^ ^,^ ^48^ ^,^ ^50^ ^,^ ^54^ ^,^ ^56^. In addition, questioning the possibility of improvement associated with this frustration is also noticed: “*How much can I help them? They’ve had this disease for months, if not years, and they’re going to have this for the rest of their lives*” ^50^ (p. e39), showing the difficulty of remaining hopeful in this context: “*Trying to sustain hope and remembering that recovery is possible...* (...) *can be quite challenging*” ^38^ (p. 7).

The complexity and severity involved in the cases of the analyzed studies, as well as the difficulty in forming and maintaining a good therapeutic relationship with the patients are added to the various variables involved in dealing with any individual, such as: culture ^39^ ^,^ ^46^, gender ^47^, comorbidities with other conditions ^51^, or care at specific moments in their life ^41^ ^,^ ^53^ ^,^ ^55^. These specificities bring another perspective shown by the studies: the importance of individualized care, one that is tailored to each patient ^38^ ^,^ ^40^ ^,^ ^41^ ^,^ ^42^ ^,^ ^44^ ^,^ ^45^ ^,^ ^46^ ^,^ ^47^ ^,^ ^48^ ^,^ ^51^ ^,^ ^52^ ^,^ ^53^ ^,^ ^56^. Some speeches by professionals show this idea: “*treatment needs to be tailored accordingly, to meet individual needs through a personal treatment plan that is focused on the person rather than the diagnosis*” ^56^ (p. 395).

### Theme 2: Reflecting on treatment: relevance of discussion, communication, and flexibility in health professionals’ work with anorexia nervosa/bulimia nervosa

This topic is about the experiences of professionals in health services, especially regarding their work experiences with a specialized multidisciplinary health team, as well as in the daily practices of care in the services based on the protocols and available resources. The indispensability of working in a multidisciplinary team is highlighted when mentioning the difficulties of the care scenario. On the other hand, the difficulty of communication between professionals of the same team or between different professionals of the health system seems to be an obstacle to a good service to service users.

Many studies ^38^ ^,^ ^39^ ^,^ ^41^ ^,^ ^44^ ^,^ ^45^ ^,^ ^48^ ^,^ ^50^ ^,^ ^53^ ^,^ ^56^ have highlighted the importance of a multidisciplinary team in the health services responsible for providing care to people with eating disorders, covering all aspects of the patient (psychological, physical, and social), in a “*diverse team with diverse skills*” ^38^ (p. 8), which work with coordinated actions in decision making. “*It’s the division of the responsibilities* (...) *where I feel confident to be able to talk with families and explain*” ^50^ (p. e38), so that “*we decide together*” ^45^ (p. 5).

Other publications also point out that working in a team can help to overcome the difficulties found in working with eating disorder patients with tips, supervision, and observing the more experienced ^44^ ^,^ ^48^: “*talking to my other consultant colleagues, especially with regard to approaching family and conflicts*” ^44^ (p. 4). However, other articles ^40^ ^,^ ^50^ also highlighted barriers found in teamwork such as communication difficulties and the fact that professionals do not feel comfortable asking questions, making dialogue difficult: “*sometimes I didn’t feel that comfortable in asking questions*” ^43^ (p. 684).

Communication obstacles are also present between different health centers: between general practitioners/pediatricians and specialized services or in the communication between two different specialized services in moments of transition ^38^ ^,^ ^41^ ^,^ ^45^ ^,^ ^53^. In the experience of professionals, they should work together so that better care provided to users is possible. One professional pointed out: “*... other times you don’t hear back from people, and that can be really difficult*” ^41^ (p. 447).

Other impasses in the daily practice of health services are related to the structures of the places where the services are located: hospitals, clinics, and specialized outpatient clinics. For example, the lack of financial resources, the great demand for services, reducing the time dedicated to each patient, and the challenges to find an adequate setting or lack of specialized professionals ^38^ ^,^ ^41^ ^,^ ^42^ ^,^ ^50^ ^,^ ^56^. Some speeches by professionals underline the lack of funding: “*I think one of the problems is when the funding runs out*” ^56^ (p. 394); “*More funding for more staff would be the best to be able to provide more care for more patients*” ^38^ (p. 8).

The overload of the services, and consequently of the professionals, also leads the multidisciplinary team to lack time for study, dedication, and reflection about the work carried out ^39^ ^,^ ^41^ ^,^ ^42^ ^,^ ^56^. This appears in reports of several studies: “*I don’t know if its linking gaps in my knowledge or if it’s the time to think that might be more of what’s missing sometimes*” ^39^ (p. 425).

Some healthcare institutions have stricter diagnostic guidelines, protocols, and improvement criteria. In some studies, workers experienced the protocols or guides as support in facing doubts, thus facilitating care practice ^41^ ^,^ ^44^ ^,^ ^48^ ^,^ ^50^ ^,^ ^56^, giving consistency: “*It’s definitely a disease process that needs consistency and regular planning*” ^50^ (p. e39), as well as standardized conducts: “*It might be helpful to have some kind of training program or something that would mean everyone was going out to the families and doing the same thing*” ^48^ (p. 34).

On the other hand, others experienced the protocols and guides as instruments with parameters that are too rigid, which makes the practice inflexible and limits the possibilities of adjusting to the needs of each one ^39^ ^,^ ^40^ ^,^ ^45^ ^,^ ^46^ ^,^ ^47^ ^,^ ^53^ ^,^ ^54^. “*The actual clinical work wanted diversity. They want to use different models*” ^39^ (p. 425); “*my fear is that there is a guide, but people are individuals*” ^40^ (p. 730).

Differential diagnosis was also mentioned in the studies retrieved, and doubts were reported in formulating diagnostic criteria, especially when there are comorbidities or in the case of atypical anorexia nervosa ^40^ ^,^ ^52^ ^,^ ^53^ ^,^ ^54^. Some of the study participants stated that “*patients’ weight behavior and family circumstances are presented as uncertain evidence of diagnosis*” ^54^ (p. 462); and that they feel “*uneasy and unsure about the implications of using a fixed set of criteria recovery*” ^40^ (p. 731).

Personal resources that help in the daily practice of health services were also mentioned, such as previous knowledge and experience in the area, making them confident in providing interventions ^43^ ^,^ ^46^ ^,^ ^47^ ^,^ ^48^. “*I feel more confident knowing the physical, medical side*” ^47^ (p. 4). Other interventions that have proved successful in the perception of professionals are practices with groups ^43^ ^,^ ^46^ ^,^ ^47^ ^,^ ^53^ ^,^ ^56^ providing mutual support among patients ^38^, and those aimed at the development of daily skills, promoting the development of autonomy ^38^ ^,^ ^48^ ^,^ ^49^ ^,^ ^50^ ^,^ ^51^ ^,^ ^52^ ^,^ ^53^.

### Theme 3: Dealing with ambivalences: experiences of health professionals with family members of people with anorexia nervosa/bulimia nervosa in the therapeutic context

This theme deals with the experience of professionals with family and companions of patients treated at the health service and the importance of this support network for the treatment. Despite recognizing the importance of family members of people with anorexia nervosa/bulimia nervosa, the participants in the analyzed studies also felt that they can contribute to maintaining symptoms, hindering, above all, the accountability and autonomy of patients. Therefore, they demonstrate ambivalent feelings about working with family members.

Many professionals who participated in the recovered studies reinforced the importance of the family actively participating in the treatment, seeing it as “crucial” and even “central” ^38^ ^,^ ^41^ ^,^ ^42^ ^,^ ^43^ ^,^ ^44^ ^,^ ^45^ ^,^ ^48^ ^,^ ^50^ ^,^ ^51^ ^,^ ^52^ ^,^ ^53^ ^,^ ^55^, since “*they play such a key role*” ^43^ (p. 684). One professional said: “*They’re going to be the ones to keep an eye out for warning signs. So, it’s important to involve them in the care as well*” ^44^ (p. 5).

On the other hand, the family can be seen by these workers as an obstacle to treatment due to their controlling attitudes and often symbiotic patterns of attachment, making it difficult for patients to take responsibility and develop autonomy, and often contribute to the maintenance of symptoms ^38^ ^,^ ^39^ ^,^ ^44^ ^,^ ^45^ ^,^ ^48^ ^,^ ^50^ ^,^ ^53^ ^,^ ^54^ ^,^ ^55^. One professional says that: “*With one patient, I felt the family was contributing to the ED* [eating disorder]” ^50^ (p. e39). Studies which investigated the transition of patients from pediatric to adult outpatient clinics ^55^ and the entry of people with eating disorders into college ^41^ report on the parents’ difficulty in dealing with their children, who start to have greater legal control over the decisions made, and the anxieties arising from these moments of growth and separation: “*This has been a big issue where parents have gotten together and said even though they are 18 we can still make decisions*” ^55^ (p. 764).

The professionals participating in the studies therefore recognize that the family is essential in the treatment, but can be a factor which contributes to maintain the chronicity of symptoms. This reinforces that eating disorders care services must include the family in the therapeutic plan. In the words of a professional: “*we are bringing the family closer together as well and, kind of, empowering them to support the patient*” ^38^ (p. 8). One study also mentions the relevance of looking at family members as people who are cared for: “*I think that as a service we have to support the families because their lives really do change*” ^41^ (p. 449).

In view of the results explained in the previous descriptive themes, two analytical themes were developed.

### Analytical theme 1: Making work with eating disorders palatable: malleability necessary for health professionals in bonding with anorexia nervosa/ bulimia nervosa patients and their families

According to the analyzed studies, working with patients with anorexia nervosa/bulimia nervosa is a challenging experience and unpalatable from the professionals’ reports. In leaving their comfort zone, workers are faced with the rigidity of these patients and the tenacity with which they cling to the symptoms, not recognizing the eating disorder as a problem, not seeking help, and not collaborating with communication. This scenario leads to feelings of frustration, insufficiency, fear, and ambivalence, among others. In this analytical theme, we emphasize that the difficult feelings that these patients and their families place on professionals require flexibility to deal with the difficulties imposed by the clinical encounter and seek hope, regardless of the obstacles.

The professionals showed that flexibility was necessary because of communication difficulties, seeking the best way to talk to patients, as well as dealing with their own feelings when in the contact with the users of the health services. Malleability can be an adaptive way for professionals to deal with the typical rigidity of these patients, promoting greater openness to the therapeutic alliance. From these studies, we can see that a lot of flexibility in clinical management is also needed when dealing with the many variables of each unique patient and with family difficulties. Considering the impasses within the care scenario, professionals may have protocols and guidelines, but they had to adapt them to each case, work as a team to think about the complexity of each person assisted/treated, with flexibility to work with other professionals or even other services. However, this malleability presupposes that professionals are guaranteed minimum working conditions, such as human and technological resources, time for study, discussion, and adequate care and training.

### Analytical theme 2: Leaving the professional comfort zone: transition from multi to interdisciplinary

Caring for people with anorexia nervosa/bulimia nervosa requires professionals to leave their known world and be flexible when dealing with patients and their families. However, they still cling to the comfort zone of staying within their own specialization. In the experience of the professionals participating in the studies, it is possible to perceive that there is an intense difficulty in dialogue because there is no integration between the disciplines, there is no openness to the various new skills necessary when working with eating disorders.

Therefore, although the professionals of the analyzed studies emphasize the importance of a multidisciplinary team, in practice they do not function as interdisciplinary. This brings obstacles to creating knowledge in practice, which takes place during care in a different way from what they learned before. This lack of interdisciplinarity may be another factor that has implications for the fact that working with eating disorders is considered difficult and “unpalatable”, and potentially for the difficulty of seeing family members as a target population of care.

## Discussion

This review allowed us to synthesize qualitative evidence on the experience of health professionals in caring for people with anorexia and bulimia nervosa. Three descriptive themes were developed considering the intense and difficult experience of working with this type of demand which requires great flexibility from professionals, leaving their comfort zone, both in terms of caring for the patient and their families, as well as in facing a care scenario within the health services which does not always favor the professional’s work.

The specialized literature in the area of ​​eating disorders ^16^ ^,^ ^17^ ^,^ ^18^ shows us that although the therapeutic alliance is essential to treatment, it is considered an achievement which is built on unsafe terrain, bringing fragility and instability in the professional-patient relationship. The primary qualitative data gathered herein are congruent with previous literature review studies, demonstrating this difficulty in contacting patients ^18^ ^,^ ^19^ and their families ^26^.

The justifications offered by the professionals of the studies analyzed herein include: the complex and multifactorial characteristics of the eating disorders; non-recognition of the need for help on the part of the patient; the difficulty of communication ^38^ ^,^ ^40^ ^,^ ^42^ ^,^ ^43^ ^,^ ^44^ ^,^ ^45^ ^,^ ^46^ ^,^ ^47^ ^,^ ^48^ ^,^ ^50^ ^,^ ^54^ ^,^ ^55^ ^,^ ^56^; and non-recognition of eating disorder as a disease ^39^ ^,^ ^41^ ^,^ ^42^ ^,^ ^43^ ^,^ ^44^ ^,^ ^45^ ^,^ ^47^ ^,^ ^54^ ^,^ ^55^, all of which are in line with other previous reviews ^18^ ^,^ ^19^. This shows us that there is already some acknowledgement to these obstacles and difficulties. However, what are the resources spent by professionals to deal with this unpleasant scenario that raises so many difficult feelings?

This first analytical theme brings us clues to its answer. People with anorexia nervosa/bulimia nervosa have very rigid personality characteristics, with little control over impulsivity and high intensity of emotional reactions. Which may also be the reason for some of their sociability difficulties ^3^ ^,^ ^6^. Therefore, people with eating disorders often experience considerable adversity in interpersonal relationships, including with health professionals ^7^ ^,^ ^15^.

Participants in the analyzed studies found themselves forced to leave their comfort zone and deal with this rigidity in a malleable way. According to the investigations ^42^ ^,^ ^43^ ^,^ ^44^ ^,^ ^47^ ^,^ ^49^ ^,^ ^50^ ^,^ ^51^ ^,^ ^54^, professionals demonstrate care and parsimony in their dialogue with patients with anorexia nervosa/bulimia nervosa. An ability to position oneself in a more open and intimate way has the potential to favor the openness of patients who perceive the availability of the professional as favoring the therapeutic alliance ^57^ ^,^ ^58^.

With this careful and open attitude towards the other - going beyond the adjustment of a protocol or seeking a “success” and “improvement” factor in the treatment - emotional frankness and availability to be with patients has the potential to be a factor that facilitates the bond ^16^ ^,^ ^59^, allowing for a more favorable outcome in the treatment ^17^. In the meantime, it is worth noting that what can be considered an improvement in the treatment of eating disorders is a topic which is still much discussed in the academic and clinical fields ^59^ ^,^ ^60^.

In the same sense, professionals in the analyzed studies ^39^ ^,^ ^40^ ^,^ ^41^ ^,^ ^45^ ^,^ ^46^ ^,^ ^47^ ^,^ ^48^ ^,^ ^50^ ^,^ ^53^ ^,^ ^54^ ^,^ ^56^ describe the availability of protocols as something that can help them, but that must also be used sparingly and malleably, since the people served have unique cultural and social variables. This data reinforces that professionals understand the relevance of healthcare thought from the uniqueness of each patient without failing to place it in its social context and the complexity that the multiple facets of illness demand ^10^ ^,^ ^61^.

Therefore, this view favors appreciating comprehensiveness in the care provided to the patient, taking into account a notion of bio-psycho-social health ^10^ ^,^ ^61^. In doing so, there is space for professionals to not only look at the person with anorexia nervosa/bulimia nervosa regarding descriptive diagnostic criteria, but with the possibility that they engage in everyday tasks and have enriched interpersonal relationships, according to some of the articles analyzed in this review ^38^ ^,^ ^41^ ^,^ ^43^ ^,^ ^46^ ^,^ ^47^ ^,^ ^48^ ^,^ ^49^ ^,^ ^50^ ^,^ ^51^ ^,^ ^52^ ^,^ ^53^ ^,^ ^56^. This point of view also reinforces care for family members since it values community engagement in health practices ^8^ ^,^ ^62^ ^,^ ^63^.

It is important to point out that this necessary malleability in dealing with patients and their families assumes that health workers are guaranteed minimum working conditions, such as human and technological resources, study time, discussions, and adequate care and training. Many of the studies analyzed ^38^ ^,^ ^39^ ^,^ ^41^ ^,^ ^42^ ^,^ ^50^ ^,^ ^56^ consider the precariousness of these conditions, which can make work more strenuous, increasing the vulnerability of professionals to illness in facing the difficult feelings cited by professionals ^20^ ^,^ ^21^.

Although the professionals in the studies that composed the corpus of this analysis reported “getting out of their comfort zone” when in contact with patients, this was little observed in regard to openness to communication and practices with other professionals, which can be an obstacle to an interdisciplinary approach, the second analytical theme of this study. Professionals recognize that they need to collaborate with the various knowledge areas involved in the care of people with anorexia nervosa/bulimia nervosa, as recommended by the specialized literature ^6^. Nevertheless, the research gathered in this review indicates that professionals find it difficult to maintain dialogue with other areas or they feel insecure when doing so ^42^ ^,^ ^43^ ^,^ ^44^ ^,^ ^47^ ^,^ ^49^ ^,^ ^50^ ^,^ ^51^ ^,^ ^54^.

As we can see in this study, other studies reaffirm the obstacles faced by physicians in implementing interdisciplinarity ^64^ ^,^ ^65^. Authors in the area argue that the difficulty in maintaining a climate of horizontality which enables a true integration between knowledge areas refers to the legacy of the model founded in the 19th century that intensified specialization of the disciplines ^65^.

The attitude of the healthcare team, from an interdisciplinary perspective, can also favor opening the professional to the perception of the family as a target of care, not only making them responsible for treating the member diagnosed with anorexia nervosa/bulimia nervosa ^26^ ^,^ ^66^. In the described studies ^38^ ^,^ ^41^ ^,^ ^42^ ^,^ ^43^ ^,^ ^44^ ^,^ ^45^ ^,^ ^48^ ^,^ ^51^ ^,^ ^53^ ^,^ ^55^, the participants recognize that the family is essential in the treatment, while also acknowledging that they can be a factor which contributes to maintain chronic symptoms. This ambivalence can be an impediment in the view of the family member as a target of care, which also demands listening to the interdisciplinary team ^26^.

## Conclusion

This study illuminates aspects within clinical practice regarding treatment of eating disorders, which can bring relevant implications for such practices in the following senses: health professionals must be trained to favor flexibility and affective openness to bond with patients with eating disorders, understanding that the professional’s rigidity can intensify the austerity of the patients treated. The training of health workers/professionals must also raise awareness on the importance of working in an interdisciplinary way, encouraging the professional to being open to listening, transforming, and building new care actions together with other professionals, leaving the comfort zone of the discipline itself and of their own knowledge.

The comprehensive and interdisciplinary look also leads to another way of understanding the families of affected people. From this, we include a third implication of this study for health practices: the relevance of including the family in the therapeutic scenario as a care recipient, and not only in the role of caregiver. For all of this to be possible, it would be extremely important that health services are properly equipped and provided with spaces for discussion, training for the obstacles placed in the care scenario, guaranteeing minimum conditions for health workers.

Despite the rigorous methodological procedure, this study has some limitations. The corpus analysis did not include Latin American publications. From the point of view of the methodological quality of the analyzed studies, it was observed that there are flaws in the reflexivity criterion, meaning that several studies do not explain the position or influence on the researcher in the context of performing the investigations. Another limitation of this review regards the exclusion of grey literature, such as dissertations and theses. These aspects can illuminate the need for future studies, especially focusing on the national perspective.

## References

[1] World Health Organization ICD-11. International classification of diseases, 11th
revision..

[2] Mitchell JE, Peterson CB (2020). Anorexia nervosa. N Engl J Med.

[3] Oliveira-Cardoso EA, Santos MA, Barroso SM, Scorsolini-Comin F, Nascimento E (2019). Avaliação psicológica: contextos de atuação, teoria e modos de
fazer.

[4] American Psychiatric Association (2013). Diagnostic and statistical manual of mental disorders..

[5] Oliveira-Pereira TTS, Santos MA (2012). Care group for mental health staff a professional development
strategy. J Hum Growth Dev.

[6] Santos MA, Valdanha-Ornelas ED, Leonidas C, Oliveira-Cardoso EA, Amparo DM, Morais RAO, Brasil KT, Lazzarini ER (2020). Adolescência: psicoterapias e mediações terapêuticas na clínica dos
extremos.

[7] LaMarre A, Rice C (2021). Healthcare providers' engagement with eating disorder recovery
narratives opening to complexity and diversity. Med Humanit.

[8] Treasure J, Parker S, Oyeleye O, Harrison A (2021). The value of including families in the treatment of anorexia
nervosa. Eur Eat Disord Rev.

[9] Ferreira IMS, Souza APL, Azevedo LDS, Leonidas C, Santos MA, Pessa RP (2021). The influence of mothers on the development of their daughter's
eating disorders an integrative review. Arch Clin Psychiatry.

[10] Oliveira TTSS, Fabrici EP, Santos MA (2018). Estrutura e funcionamento de uma equipe de saúde mental de
Trieste na perspectiva de seus integrantes um estudo
qualitativo. Psicol Pesqui.

[11] Fassino S, Abbate-Daga G (2013). Resistance to treatment in eating disorders a critical
challenge. BMC Psychiatry.

[12] Sibeoni J, Orri M, Valentin M, Podlipski MA, Colin S, Pradere J (2017). Metasynthesis of the views about treatment of anorexia nervosa in
adolescents perspectives of adolescents, parents, and
professionals. PLoS One.

[13] Souza APL, Pessa RP (2016). Tratamento dos transtornos alimentares fatores associados ao
abandono. J Bras Psiquiatr.

[14] Souza APL, Valdanha-Ornelas ÉD, Santos MA, Pessa RP (2019). Significados do abandono do tratamento para pacientes com
transtornos alimentares. Psicol Ciênc Prof.

[15] Fleming C, Le Brocque R, Healy K (2021). How are families included in the treatment of adults affected by
eating disorders A scoping review. Int J Eat Disord.

[16] Ramos TMB, Pedrão LJ (2013). Acolhimento e vínculo em um serviço de assistência a portadores
de transtornos alimentares. Paidéia (Ribeirão Preto).

[17] Werz J, Voderholzer U, Tuschen-Caffier B (2022). Alliance matters but how much? A systematic review on therapeutic
alliance and outcome in patients with anorexia nervosa and bulimia
nervosa. Eat Weight Disord.

[18] Seah XY, Tham XC, Kamaruzaman NR, Yobas P (2017). Attitudes and challenges of healthcare professionals managing
people with eating disorders a literature review. Arch Psychiatr Nurs.

[19] Thompson-Brenner H, Satir DA, Franko DL, Herzog DB (2012). Clinician reactions to patients with eating disorders a review of
the literature. Psychiatr Serv.

[20] Brolese DF, Lessa G, Santos JLG, Mendes JS, Cunha KS, Rodrigues J (2017). Resilience of the health team in caring for people with mental
disorders in a psychiatric hospital. Rev Esc Enferm USP.

[21] Monteiro DT, Mendes JMR, Beck CLC (2019). Health professionals' mental health a look at their
suffering. Trends Psychol.

[22] Johns G, Taylor B, John A, Tan J (2019). Current eating disorder healthcare services - the perspectives
and experiences of individuals with eating disorders, their families and
health professionals systematic review and thematic
synthesis. BJPsych Open.

[23] Babb C, Jones CRG, Fox JRE (2022). Investigating service users' perspectives of eating disorder
services a meta-synthesis. Clin Psychol Psychother.

[24] Wetzler S, Hackmann C, Peryer G, Clayman K, Friedman D, Saffran K (2020). A framework to conceptualize personal recovery from eating
disorders a systematic review and qualitative meta-synthesis of perspectives
from individuals with lived experience. Int J Eat Disord.

[25] Fox JR, Dean M, Whittlesea A (2017). The experience of caring for or living with an individual with an
eating disorder a meta-synthesis of qualitative studies. Clin Psychol Psychother.

[26] Gil M, Simões MM, Oliveira-Cardoso EA, Pessa RP, Leonidas C, Santos MA (2022). Percepção de familiares de pessoas com transtornos alimentares
acerca do tratamento uma metassíntese da literatura. Psicol Teor Pesqui.

[27] Simões M, Santos MA (2021). Paternity and parenting in the context of eating disorders an
integrative literature review. Psicol Teor Pesq.

[28] Polita NB, Alvarenga WA, Leite ACAB, Araújo JS, Santos LBPA, Zago MMF (2018). Care provided by the father to the child with cancer under the
influence of masculinities qualitative meta-synthesis. Rev Bras Enferm.

[29] Critical Appraisal Skills Programme CASP checklist: 10 questions to help you make sense of qualitative
reasearch..

[30] National Institute for Health and Care Research PROSPERO. International prospective register of systematic
reviews..

[31] Tong A, Flemming K, McInnes E, Oliver S, Craig J (2012). Enhancing transparency in reporting the synthesis of qualitative
research ENTREQ. BMC Med Res Methodol.

[32] Cooke A, Smith D, Booth A (2012). Beyond PICO the SPIDER tool for qualitative evidence
synthesis. Qual Health Res.

[33] Ouzzani M, Hammady H, Fedorowicz Z, Elmagarmid A (2016). Rayyan-a web and mobile app for systematic
reviews. Syst Rev.

[34] Woods M, Paulus T, Atkins DP, Macklin R (2015). Advancing qualitative research using qualitative data analysis
software (QDAS) Reviewing potential versus practice in published studies
using ATLAS.ti and NVivo, 1994-2013. Soc Sci Comput Rev.

[35] Viera A, Garrett J (2005). Understanding interobserveragreement the Kappa
statistic. Fam Med.

[36] Page MJ, McKenzie JE, Bossuyt PM, Boutron I, Hoffmann TC, Mulrow CD (2021). The PRISMA 2020 statement an updated guideline for reporting
systematic reviews. BMJ.

[37] Thomas J, Harden A (2008). Methods for the thematic synthesis of qualitative research in
systematic reviews. BMC Med Res Methodol.

[38] Webb H, Dalton B, Irish M, Mercado D, McCombie C, Peachey G (2022). Clinicians' perspectives on supporting individuals with severe
anorexia nervosa in specialist eating disorder intensive treatment
settings. J Eat Disord.

[39] Kanakam N (2021). Therapists' experiences of working with ethnic minority females
with eating disorders a qualitative study. Cult Med Psychiatry.

[40] McDonald S, Williams AJ, Barr P, McNamara N, Marriott M (2021). Service user and eating disorder therapist views on anorexia
nervosa recovery criteria. Psychol Psychother.

[41] Webb H, Schmidt U (2021). Facilitators and barriers to supporting young people with eating
disorders during their transition to, and time at, university an exploration
of clinicians' perspectives. Eur Eat Disord Rev.

[42] Wu W, Chen S (2021). Nurses' perceptions on and experiences in conflict situations
when caring for adolescents with anorexia nervosa a qualitative
study. Int J Mental Health Nurs.

[43] Farrington A, Huntley-Moore S, Donohue G (2020). "I found it daunting": an exploration of educational needs and
experiences of mental health student nurses working with children and
adolescents with eating disorders.. J Psychiatr Ment Health Nurs.

[44] Davidson AR, Braham S, Dasey L, Reidlinger DP (2019). Physicians' perspectives on the treatment of patients with eating
disorders in the acute setting. J Eat Disord.

[45] Dimitropoulos G, Kimber M, Singh M, Williams EP, Loeb KL, Hughes EK (2019). Stay the course practitioner reflections on implementing
family-based treatment with adolescents with atypical
anorexia. J Eat Disord.

[46] Holmes S (2018). The role of sociocultural perspectives in eating disorder
treatment a study of health professionals. Health (London).

[47] Kinnaird E, Norton C, Tchanturia K (2018). Clinicians' views on treatment adaptations for men with eating
disorders a qualitative study. BMJ Open.

[48] Watt J, Dickens GL (2018). Community-based mealtime management for adolescents with anorexia
nervosa a qualitative study of clinicians' perspectives and
experiences. J Child Adolesc Psychiatr Nurs.

[49] Wehrens R, Walters BH (2018). Understanding each other in the medical encounter exploring
therapists' and patients' understanding of each other's experiential
knowledge through the Imitation Game. Health (London).

[50] Harken W, Maxwell J, Hainline M, Pollack L, Roberts C (2017). Perceptions of caring for adolescents with eating disorders
hospitalized on a general pediatric unit. J Pediatr Nurs.

[51] Kinnaird E, Norton C, Tchanturia K (2017). Clinicians' views on working with anorexia nervosa and autism
spectrum disorder comorbidity a qualitative study. BMC Psychiatry.

[52] Cruzat MC, Aspillaga HC, Behar AR, Espejo LMC, Gana HC (2013). Facilitadores de la alianza terapéutica en la anorexia nerviosa
una mirada desde la diada terapeuta-paciente. Rev Chil Neuro-Psiquiatr.

[53] Dimitropoulos G, Tran AF, Agarwal P, Sheffield B, Woodside B (2013). Challenges in making the transition between pediatric and adult
eating disorder programs a qualitative study from the perspective of service
providers. Eat Disord.

[54] Hunt D, Churchill R (2013). Diagnosing and managing anorexia nervosa in UK primary care a
focus group study. Fam Pract.

[55] Dimitropoulos G, Tran AF, Agarwal P, Sheffield B, Woodside B (2012). Navigating the transition from pediatric to adult eating disorder
programs perspectives of service providers. Int J Eat Disord.

[56] Reid M, Williams S, Burr J (2010). Perspectives on eating disorders and service provision a
qualitative study of healthcare professionals. Eur Eat Disord Rev.

[57] Simonds LM, Spokes N (2017). Therapist self-disclosure and the therapeutic alliance in the
treatment of eating problems. J Eat Disord.

[58] Zugai JS, Stein-Parbury J, Roche M (2018). Therapeutic alliance, anorexia nervosa and the inpatient setting
a mixed methods study. Int J Adv Nurs.

[59] Souza LV, Santos MA (2015). Histórias de sucesso de profissionais da saúde no tratamento dos
transtornos alimentares. Psicol Ciênc Prof.

[60] Miller PM (1996). Redefining success in eating disorders. Addict Behav.

[61] Oliveira ALM, Peres RS (2021). As oficinas terapêuticas e a lógica do cuidado psicossocial
concepções dos(as) coordenadores(as). Psicol Ciênc Prof.

[62] Marcon TD, Girz L, Stillar A, Tessier C, Lafrance A (2017). Parental involvement and child and adolescent eating disorders
perspectives from residents in psychiatry, pediatrics, and family
medicine. J Am Acad Child Adolesc Psychiatry.

[63] Peckmezian T, Paxton SJ (2020). A systematic review of outcomes following residential treatment
for eating disorders. Eur Eat Disord Rev.

[64] Derriennic J, Barais M, Goff DL, Fernandez G, Borne FL, Reste JYL (2021). Patient, carer and healthcare professional experiences of complex
care quality in multidisciplinary primary healthcare centres qualitative
study with face-to-face, in-depth interviews and focus groups in five French
multidisciplinary primary healthcare centres. BMJ Open.

[65] Walton V, Hogden A, Long JC, Johnson JK, Greenfield D (2019). How do interprofessional healthcare teams perceive the benefits
and challenges of interdisciplinary ward rounds. J Multidiscip Healthc.

[66] Siqueira ABR, Santos MA, Leonidas C (2020). Confluências das relações familiares e transtornos alimentares
revisão integrativa da literatura. Psicol Clín.

